# Evaluation of New Fluorescent Lipophosphoramidates for Gene Transfer and Biodistribution Studies after Systemic Administration

**DOI:** 10.3390/ijms161125941

**Published:** 2015-11-02

**Authors:** Nawal Belmadi, Mathieu Berchel, Caroline Denis, Wilfried Berthe, Yann Sibiril, Tony Le Gall, Jean-Pierre Haelters, Paul-Alain Jaffres, Tristan Montier

**Affiliations:** 1 Unité INSERM 1078, Faculté de Médecine, Université de Bretagne Occidentale, Université Européenne de Bretagne, 22 avenue Camille Desmoulins, 29238 Brest cedex 3, France; nawal.belmadi@univ-brest.fr (N.B.); caroline.denis@chu-brest.fr (C.D.); yann.sibiril@univ-brest.fr (Y.S.); tony.legall@univ-brest.fr (T.L.G.); 2 Plateforme SynNanoVect, Biogenouest, SFR 148 ScInBioS, Université de Bretagne Occidentale, Faculté de Médecine, 22 rue Camille Desmoulins, 29238 Brest cedex 3, France; mathieu.berchel@univ-brest.fr (M.B.); wilfried.berthe@univ-brest.fr (W.B.); Jean-Pierre.Haelters@univ-brest.fr (J.-P.H.); paul-alain.jaffres@univ-brest.fr (P.-A.J.); 3 UMR CNRS 6521, Université de Bretagne Occidentale, Université Européenne de Bretagne, Faculté des Sciences, 6 avenue Victor Le Gorgeu, 29238 Brest, France; 4 Laboratoire de génétique moléculaire et d’histocompatibilité, CHRU de Brest, 5 Avenue du Maréchal Foch, 29609 Brest cedex, DUMG, Université de Bretagne Occidentale, Université Européenne de Bretagne, 22 rue Camille Desmoulins, CS 93837-29238 Brest cedex 3, France

**Keywords:** cationic lipids, administration way, *in situ* biodistribution, *in vivo* fluorescence, pegylated formulations

## Abstract

The objective of lung gene therapy is to reach the respiratory epithelial cells in order to deliver a functional nucleic acid sequence. To improve the synthetic carrier’s efficacy, knowledge of their biodistribution and elimination pathways, as well as cellular barriers faced, depending on the administration route, is necessary. Indeed, the *in vivo* fate guides the adaptation of their chemical structure and formulation to increase their transfection capacity while maintaining their tolerance. With this goal, lipidic fluorescent probes were synthesized and formulated with cationic lipophosphoramidate KLN47 (KLN: Karine Le Ny). We found that such formulations present constant compaction properties and similar transfection results without inducing additional cytotoxicity. Next, biodistribution profiles of pegylated and unpegylated lipoplexes were compared after systemic injection in mice. Pegylation of complexes led to a prolonged circulation in the bloodstream, whereas their *in vivo* bioluminescent expression profiles were similar. Moreover, systemic administration of pegylated lipoplexes resulted in a transient liver toxicity. These results indicate that these new fluorescent compounds could be added into lipoplexes in small amounts without perturbing the transfection capacities of the formulations. Such additional properties allow exploration of the *in vivo* biodistribution profiles of synthetic carriers as well as the expression intensity of the reporter gene.

## 1. Introduction

As for any drug, the main role of nanocarriers is to provide a selective safe and efficient delivery. This allows the use of a small amount of active compound, thereby limiting the potential side effects of the drug. Tolerance is also dependent on a safe carrier elimination once the therapeutic molecule has been delivered. In order to develop effective non-viral gene transfer systems, biodistribution profiles should be investigated to better understand the *in situ* fate of these molecules and the biological barriers they have to overcome according to the route of administration. All these data are important to fine tune and design original chemical structures and optimal combinations between these different molecules. Among the present technologies, there exist some non-invasive tools such as bioluminescence and biofluorescence that can be used to track the tagged complexes while evaluating their transfection capacity [1]. Biofluorescence imaging (BFI) is a simple, low-cost and non-invasive technique. It can be carried out on living animals for different time periods, but it is important to note that the absorption and diffusion of the photons depend on the tissues depth, impacting observation [2,3]. In addition, an excitation source is needed, and can sometimes cause autofluorescence, phototoxicity or photobleaching phenomena [4]. Fluorescence technology also requires the use of fluorochromes such as cyanine, naphthalimide, NBD (7-Nitrobenz-2-oxa-1,3-diazol-4-yl), fluorescein and many others, that can be used alone or coupled to different compounds like synthetic carriers [5,6,7,8,9] or peptides [10]. Among non-viral vectors, cationic lipids are the most commonly used, and some are currently employed in gene therapy clinical trials [11,12,13,14].

Over the last decade, our laboratory has developed some original synthetic vectors and we investigated their potential as gene delivery systems. Thus, different cationic lipids such as lipophosphonates [15,16,17] and lipophosphoramidates [12,13,18,19,20,21] have been produced, and their high ability to transfect cells in culture and lungs mice were demonstrated [13,22,23,24,25,26,27]. However, the plasmid type [27,28,29], the vector [18,26,29,30,31,32] as well as the nucleic acids (NA) or synthetic carrier’s labeling techniques [8,30,33,34,35], impact greatly the distribution in tissues as well as the transgene expression. Labeling liposomes with a fluorescent probe appears as a relevant procedure to establish biodistribution profiles of lipoplexes and their pharmacokinetics, irrespective of administration route [26,27,31,32,33,34,36,37,38]. In that sense, fluorescent lipophosphoramidates bearing a single fluorophore localized in the polar head domain were synthesized [8]. Indeed, the fluorophore (NBD, cyanine or naphtalimide) was coupled with a hydrophobic part that can be associated with other lipidic compounds. These neutral fluorescent compounds were thus included at a 5% molar ratio with a lipophosphoramidate (KLN47) to produce a fluorescent liposomal solution [8]. They were incorporated in small proportions to prevent perturbations of the supramolecular assembly as well as potential modifications of the transfection properties [8].

In the present study, we report the *in vivo* biodistribution profiles after systemic administration using new fluorescent lipophosphoramidate-based formulations. In parallel, to refine our understanding of the *in situ* fate of such complexes, we also monitored their effectiveness and tolerance. Liposomal formulations were first characterized by dynamic scattering and zeta potential measurements. Physicochemical results showed that resulting formulations exhibited a size in the same range as that obtained with the lipophosphoramidate KLN47 alone. Considering zeta potential measurements, all the formulations presented an equivalent positive charge. Additionally, fluorescent lipid (FL) did not modify the DNA complexation ability of the resulting complexes. In cell lines, their transfection abilities were quite similar. Considering *in vivo* studies, liposomes and lipoplexes formulated in the presence of DSPE-PEG_2000_ (1,2-Distearoyl-sn-Glycero-3-phosphoethanolamine-*N*-[Methoxy(Polyethyleneglycol)-2000] (Ammonium salt)) exhibited a superior bioavailability during the 24 h following the injection in comparison with their equivalent formulations without steric stabilizer. However, bioluminescence imaging (BLI) showed an equivalent luciferase expression in the lungs, regardless of the presence or absence of DSPE-PEG_2000_. All together, the results indicate that these novel kinds of fluorescent compounds could be easily added to liposomal formulations to track lipoplexes *in vivo*.

## 2. Results and Discussion

### 2.1. Fluorescent Formulations

**Figure 1:**
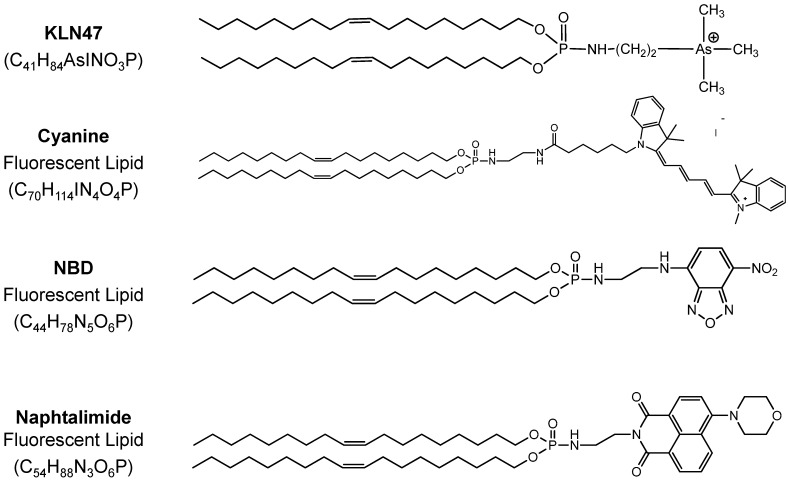
Chemical Structures of lipophosphoramidate KLN47 and fluorescent lipids.

After synthesis, the fluorescent compounds were readily incorporated into the corresponding liposomal solutions without dramatic modifications of liposomes. The chemical lipid structures are represented in Figure 1. NMR, absorbance and fluorescence spectra are represented in Supplementary data (Scheme S1). All the formulations were then characterized depending on their size and zeta potential. The results showed that liposomes size was in the same range with those obtained with the reference KLN47 (Table 1), comprised between 104 and 290 nm, except for the formulations incorporating the naphthalimide dye that exhibits a diameter around 350 nm. The zeta potential measurements were clearly positive from +46 to +66 mV for all formulations. Nevertheless, the surface charge was slightly lower for the fluorescent formulations (+45 to +50 mV) whatever the fluorochrome in comparison with the KLN47 (~+60 mV). This difference of zeta potential could be explained by the presence of the neutral fluorescent lipid in the liposomal solution. Several concentrations were tested here; the less concentrated formulations KLN47_a_ (1.3 mg/mL) were designed for *in vitro* assessments while the most concentrated solutions KLN47_b_ (10 mg/mL) and KLN47_c_ (20 mg/mL) have been produced for animal applications. For *in vivo* assays, the formulations should be more concentrated in order to reduce the volume of formulations that are then administered, thereby limiting potential toxic effects. It is important to limit the injected volume, to avoid side effects and the immune response, and to facilitate the readministration of the formulations. Different comparisons were performed. Indeed, when KLN47_a_ was compared with FL1A (FL cyanine 5 mol %), we observed a diminution in particle size and zeta potential. Considering the highly concentrated solutions, KLN47_b_ compared with FL1B and KLN47_c_ with FL1C showed a reduction of the zeta potential as well as a decrease of the particles’ size. In the end, adding such fluorescent lipid to KLN47 liposomal formulations appeared to slightly affect the chemical properties of the particle.

**Table 1:** Chemical characteristics of fluorescent cationic lipids.

Formulations	Size (nm)	Poly Index	ζ Potential (mV)
KLN47_a_—1.5 mM, 1.3 mg/mL	220.9	-	+60.2
FL1A: KLN47_a_—1.5 mM + FL Cyanine 5 mol %	189	-	+44.0
FL2A: KLN47_a_—1.5 mM + FL NBD 5 mol %	167.5	0.561	+53.1
FL2B: KLN47_a_—1.5 mM + FL NBD 10 mol %	205.0	0.418	+43.0
FL3A: KLN47_a_—1.5 mM + FL Naphtalimide 5 mol %	353.0	0.668	+50.5
FL3B: KLN47_a_—1.5 mM + FL Naphthalimide 10 mol %	354.8	0.477	+56.2
KLN47_b_—11.5 mM, 10 mg/mL	234.8	0.560	+58.4
FL1B: KLN47_b_—11.5 mM + FL Cyanine 5 mol %	141.4	0.714	+48.1
KLN47_c_—23 mM, 20 mg/mL	286.8	0.792	+65.9
FL1C: KLN47_c_—23 mM + FL Cyanine 5 mol %	164.5	0.474	+54.4

### 2.2. In Vitro Evaluation

#### 2.2.1. DNA Condensation Ability

**Figure 2:**
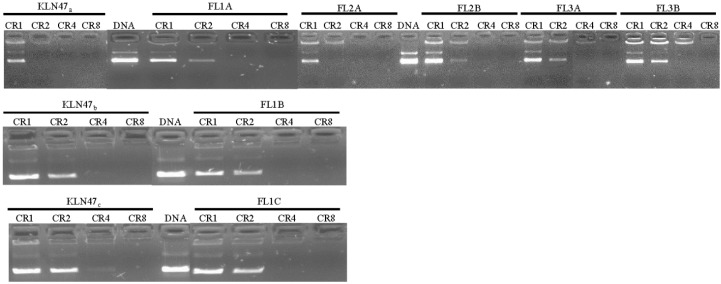
DNA binding ability of fluorescent compounds was evaluated in OptiMEM by mixing a constant mass of DNA (1 μg) with different concentrations of cationic lipids corresponding to different CRs. DNA full complexation was highlighted by the absence of DNA migration into the agarose gel, at a given CR depending on the formulations tested.

Electrophoresis migration assays on agarose gel were achieved for each lipoplex at different charge ratios (CRs), respectively 1, 2, 4 and 8. This allowed the evaluation of the ability of all the formulations to condense and protect the plasmid DNA. As mentioned above, some complexes were designed for *in vitro* experiments such as KLN47_a_, FL1A, FL2A, FL2B, FL3A and FL3B while the most concentrated, such as KLN47_b_, KLN47_c_, FL1B and FL1C were formulated for *in vivo* assays. First, ethidium bromide exclusion assays were performed to evaluate the ability of each formulation to condense plasmid DNA. Indeed, upon condensation ethidium bromide was expelled from DNA, and thus the fluorescence signal was decreased. Results showed that the different lipoplexes were able to condense the plasmid DNA with a high efficiency. The fluorescence signal rapidly decreased as the CR increased. To confirm the DNA condensation ability of each lipoplex, DNA retardation assays on agarose gel electrophoresis were next performed. Electrophoresis migration results showed that DNA condensation was partial at CR 1 and 2, and became complete at higher CRs. Globally, agarose gel retardation results (Figure 2) revealed a full condensation of pDNA at CR = 4 whatever the formulation. Thus, these lipids can efficiently complex as well as condense the pDNA. It is important to note here that the DNA condensation by the fluorescent formulation was similar to that of the KLN47. The results were in agreement with our previous reports [13,21,26]. To summarize, all formulations had an efficient DNA condensation ability at a CR of 2 or 4. The presence of a fluorescent particle did not affect the NA compaction properties of the formulations.

#### 2.2.2. *In Vitro* Transfection Assays

**Figure 3:**
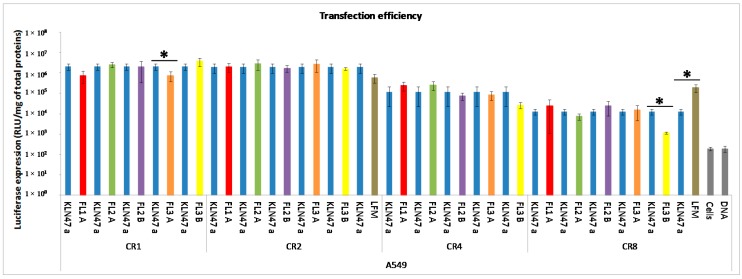

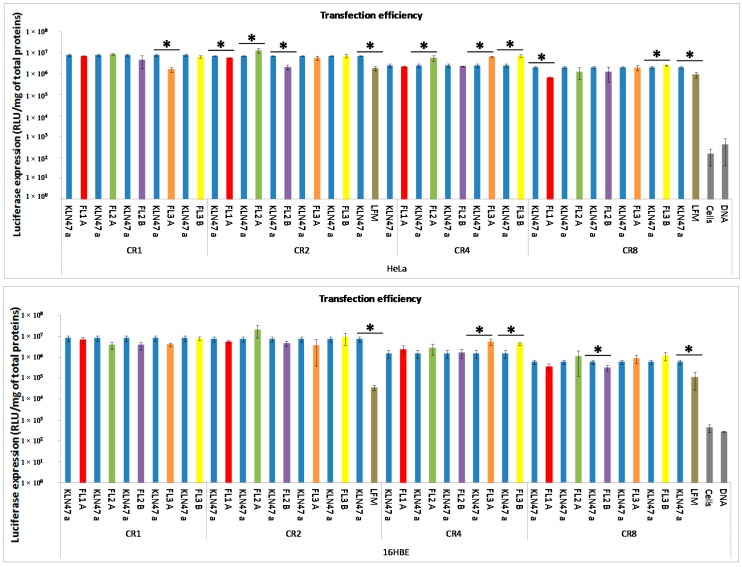
*In vitro* gene transfection efficiency of different formulations (CR ranging from one to eight) into cell lines A549, HeLa and 16HBE. The transfection procedure was performed as described in the experimental section. The luciferase expression was measured 48 hours after deposition of lipoplexes onto the cells. Results were expressed as Relative Light Units (RLU)/mg of total proteins obtained with each formulation, reflecting their transfection abilities as the mean ± SD of three wells. Asterisk (*) indicates the significance difference of gene transfection efficiency between different formulations, as determined by an ANOVA test: * *p* < 0.05.

With a view to lung gene delivery, we chose to use different cell lines (16HBE, A549 and HeLa) to evaluate the gene transfection efficiency of lipoplexes at different CRs (from 1 to 8). Agarose gel electrophoresis results suggested that the optimal condition for an efficient complexation was around CR 2 and 4. We first evaluated the reporter gene expression of the different formulations used in comparison with controls (KLN47, Lipofectamine 2000 (LFM), and naked DNA and untransfected cells). For lipofectamine 2000, the CRs have been calculated according to the manufactures recommendations. A constant mass of plasmid DNA (0.25 μg) was mixed with different concentrations of lipids (cationic lipids and lipofectamine) corresponding to the calculated CRs, whereas, the amount of lipid (cationic lipids and lipofectamine) was constant. Secondly, we assessed the impact of the CR and fluorescent probes on the transfection efficacy. The most concentrated formulations (FL1B and FL1C) were not evaluated *in vitro* because the cells died at such concentrations. Also, pegylated formulations were not tested *in vitro*, because the polyethylene glycol (PEG) was well known to reduce significantly the transfection efficiency [39]. Indeed, PEG hid the positive charges carried by the complexes, consequently reducing the electrostatic interactions between the complexes and their targeted cells. PEG is used to stabilize the complex *in vivo* and thereby reduce their early elimination. The efficient transfer of the gene of interest is ensured through cationic lipids. Regarding the transfection results represented in Figure 3, whatever the cell line, all formulations exhibited higher luciferase expression levels than the lipofectamine and naked pDNA. The fluorescent lipoplexes showed a transfection efficiency of the same magnitude when compared with KLN47 based complexes. The optimal transfection ability was obtained with a CR of 2, for all cell lines. These results were correlated to the pDNA retardation assays. The transfection efficiency was cell line dependent, and strongly decreased at CR = 8. For *in vitro* transfection efficiency and cytotoxicity, an ANOVA test was performed to evaluate the difference between formulations. All fluorescent compounds were compared to the lipophosphoramidate KLN47a used as a standard. Results showed that, concerning A549 cells, all formulations were effective for gene delivery at a CR of 2, with no significant difference with the KLN47a, except for FL3A (CR1) and FL3B (CR8). For HeLa cells, three compounds (FL2A, FL3A and FL3B) appears to be more effective than KLN47a, at CR = 4. FL2A was also more effective at a CR of 2 to transfect HeLa cells. Concerning 16HBE cells, two compounds (FL3A and FL3B) were identified as the most efficient carriers at a CR = 4. All together, these results indicated that the incorporation of these fluorescent compounds to lipophosphoramidate did not impact the transfection properties of the resulting KLN47-based formulations.

#### 2.2.3. *In Vitro* Cytotoxicity

**Figure 4:**
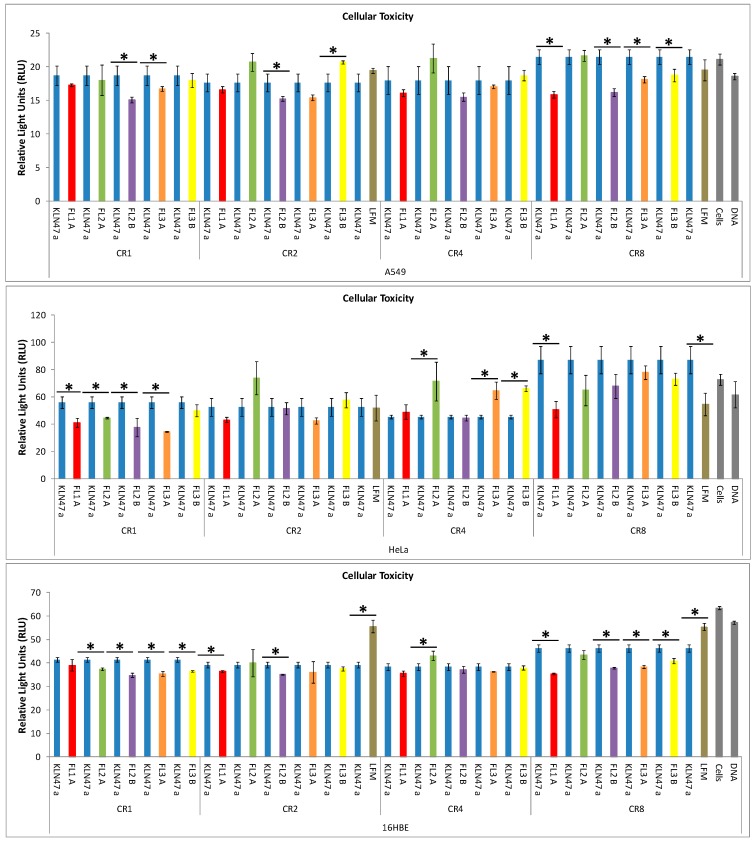
*In vitro* cytotoxicity evaluation of the different formulations into the three cell lines: A549, 16HBE and HeLa. The early cytotoxicity assessment was performed as described in experimental section, as specified by the manufacturer. The results were expressed as RLU obtained with each formulation, reflecting their cytotoxic effects as the mean ± SD of 3 wells. Asterisk (*) indicates the significance difference of gene transfection efficiency between different formulations, as determined by an ANOVA test: * *p* < 0.05.

In parallel, cytotoxic effects induced by lipoplexes were also evaluated, through measurements of adenylate kinase (AK) released into the culture medium. The presence of this enzyme in the medium was an indicator of rapid membrane injury, revealing an early cytotoxicity. As showed in Figure 4, in comparison with untreated cells, no or slight cytotoxic effect was induced by the different lipoplexes. This early cellular toxicity appeared to be CR dependent. However, the basal AK intracellular concentration was specific to each cell type and the comparison between cell lines cannot be performed using this method. Globally, the comparison between the reference and the fluorescent based-lipoplexes revealed no additional toxicity and highlighted a high tolerance of the various formulations, except for few formulations. An ANOVA test was performed to compare the different formulations. Globally, at a low CR, all formulations were not toxic whatever the cell line. Indeed, the new fluorescents compounds presented a rate of adenylate kinase similar to that obtained with untreated cells. At high CRs (CR = 8) some toxic side effects were reported. The cytotoxicity was then dose-dependent. In A549, no toxic effect was reported in comparison with KLN47a. Concerning HeLa cells, at CR = 4, FL2A, FL3A and FL3B were slightly more toxic than KLN47a. It is also reported that FL2A was a bit toxic for 16 HBE cells at a CR of 4 in comparison with a KLN47a. This low toxicity could be partially due to the presence of the fluorescent dyes into the liposomal solution.

### 2.3. In Vivo Experiments

#### 2.3.1. *In Vivo* Biodistribution

Considering *in vivo* biodistribution studies, we selected the FL1C formulation, containing high concentrated KLN47 thereby limiting the injected volume, and the cyanine dye for its compatibility with the *in vivo* imaging [40]. As previously shown, this formulation presented some interesting biophysical characteristics in terms of size (164.5 nm) and potential zeta (+54 mV). To overcome the autofluorescence of bristles, we decided to work with nude mice fed with chlorophyll-free granules. We administered the FL1C based complexes via the systemic route (mouse tail vein), and we used *in vivo* biofluorescence and bioluminescence imaging, to localize, detect and quantify the transgene (luciferase) expression. Four conditions were tested (liposomes, liposomes + DSPE-PEG_2000_, lipoplexes, and lipoplexes + DSPE-PEG_2000_), with four mice per condition. BFI was performed at 1, 3, 5 and 24 h after systemic administration, in different positions for a better identification of the fluorescent sites. As mentioned above, the intensity of the fluorescence is dependent on the animal side in front of the CCD (Charged Coupled Device) camera. As the anchorage of PEG chains is known to enhance the *in vivo* circulation time of the complexes in the bloodstream [41,42], we chose to include DSPE-PEG_2000_ at a ratio of (DSPE-PEG_2000_/pDNA) of (1:1) in some formulations.

**Figure 5:**
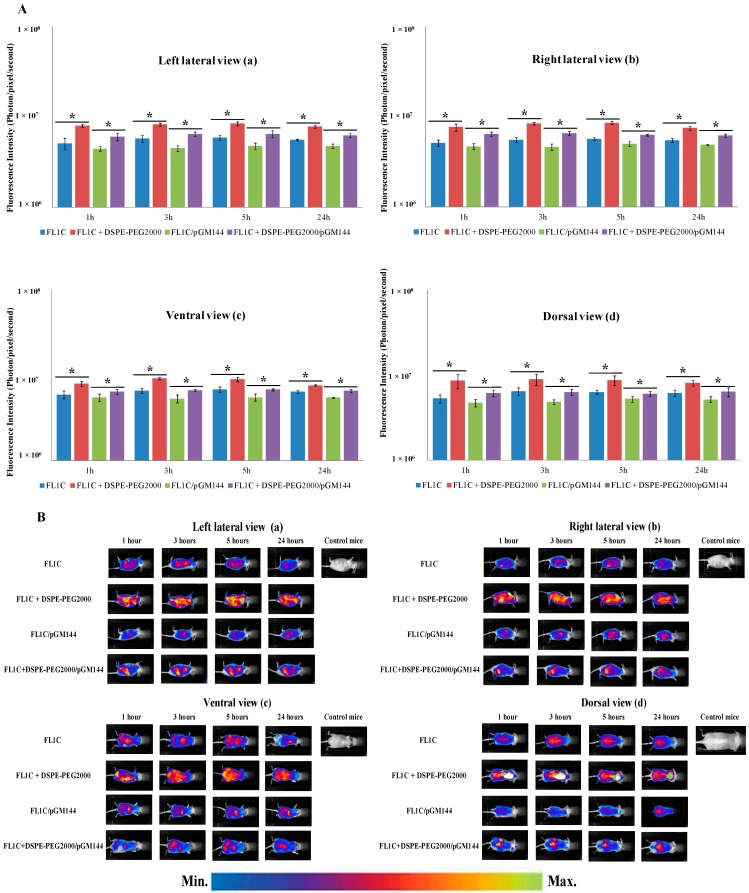

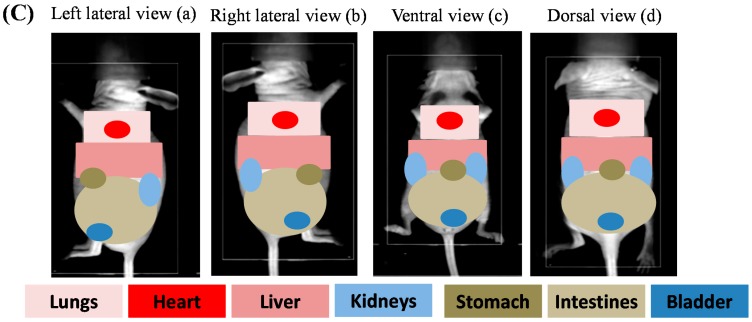
FL1C-based liposomes and lipoplexes biodistribution at different times (1, 3, 5 and 24 h). FL1C, FL1C + DSPE-PEG_2000_, FL1C/pGM144 and FL1C + DSPE-PEG_2000_/pGM144 were administered intravenously in nude mice (*n* = 4). BFI measurements and BFI images were taken in different positions (**A**,**B**); left lateral view (**a**), right lateral view (**b**), ventral view (**c**) and dorsal view (**d**) using the Nightowl II system (Berthold Technologies, Bad Wildbad, Germany) to localize all fluorescent organs, and global fluorescence intensity of FL1C-based complexes (*n* = 4) were compared (**A**). As an illustration, one mouse per experiment was represented in the different positions (**B**). In panel (**C**) was represented a schematic localization of several organs in each projection employed to clearly identify organs involved. Results were represented as photon/pixel/second reflecting the fluorescence intensity as the mean ± SD of four mice (* *p* < 0.05). The sensitivity scale of the software Winlight 32 was blocked to allow the comparison of the profiles.

One hour after injection, a significant fluorescence signal was measured, as shown in Figure 5A, B. Whatever the position of the animal and during the 24 hours post-injection for a given formulation, the fluorescence intensity was in the same order of magnitude (Figure 5A). Mice receiving liposomes and lipoplexes stabilized with DSPE-PEG_2000_ showed a higher fluorescent signal than mice receiving unpegylated liposomes or lipoplexes (*p* value < 0.05) (Figure 5). This difference between pegylated and unpegylated complexes was significant whatever the position of animal or the acquisition time. This signal remained stable and detectable in all organs at 1, 3, and 5 hours, and tended towards a decrease at 24 h (Figure 5A,B). According to Figure 5B that illustrated the whole of observations, at 1 h, the signal was localized in the whole of mice principally in the sites corresponding to the lungs, heart, liver, spleen, kidneys and urinary system. At three and five hours post administration, the fluorescent signal was concentrated in the liver, kidneys and urinary tract. At 24 h post injection whatever the formulation, the fluorescent signal was principally detected in the kidneys and urinary system (Figure 5B). Considering the FL1C + DSPE-PEG_2000_/pGM144 particles, they were rapidly accumulated in the lungs, spleen, and liver. This accretion was maintained all along the experiment. Mice receiving FL1C/pGM144 showed approximately similar but lower fluorescent intensity than those with FL1C + DSPE-PEG_2000_/pGM144. The biodistribution results of pegylated liposomes and lipoplexes indicated a higher bioavailability in comparison with the unpegylated equivalent formulations. Additionally, pegylated liposomes tended to an increased systemic circulation time compared to the lipoplexes containing PEG. As expected, the surface coating with DSPE-PEG_2000_ chains promoted the bioavailability of complexes in the blood circulation [41,42,43]. Additionally, PEG modifications prevented complexes’ aggregation, improving the stability of the formulations. Indeed, it was previously reported that pegylated complexes remained intact in the bloodstream up to three days compared to the unpegylated carriers [43]. Hydrophilic chains of PEG could avoid adsorption of the plasma proteins with the complexes by dysopsonization, limiting their degradation [37,43,44]. The PEG density on the surface of the complexes, as well as the PEG chains lengths constituted some important parameters that significantly impacted the surface properties of the particles, preventing the phagocytosis and leading to different distribution profiles [42,44,45]. The differences observed in term of fluorescence distribution after IV administration were the result of several effects, such as localization of dye into the nanocomplexes, dissociation kinetics of the complexes in the media and affinity between dyes and plasma proteins [46]. The gradual decrease of fluorescent signal was due to the elimination of the tagged compounds. Here the principal organs of elimination were the urinary tract (Figure 5B). We noticed that such fluorescent formulations were mainly eliminated after IV administration via the kidneys and not through the bilirubin pathway.

#### 2.3.2. *In Vivo* Bioluminescence Imaging

**Figure 6:**
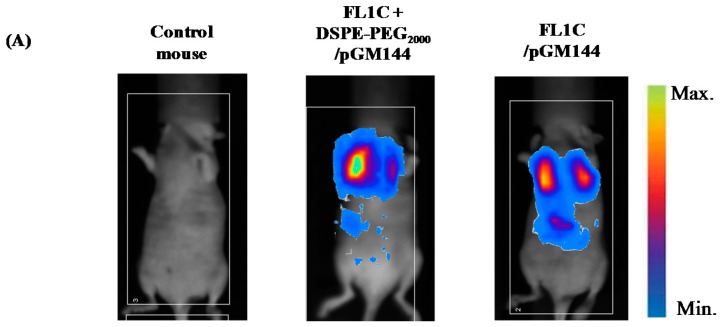

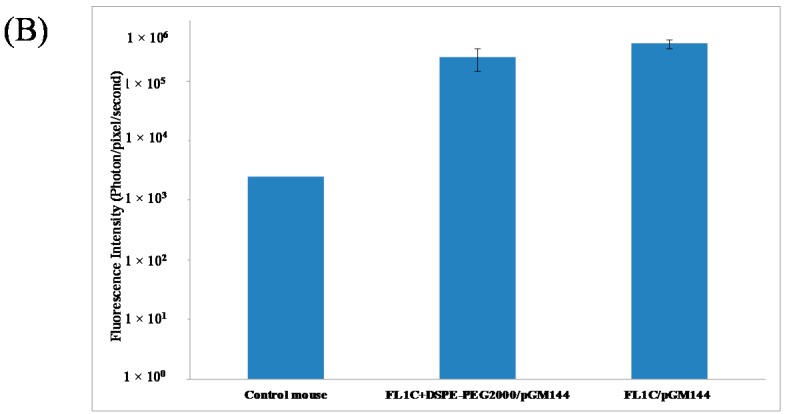
*In vivo* localization of luciferase (*luc*) expression reported at 24 h after intravenous delivery of FL1C-based lipoplexes (**A**); and Bioluminescence quantification (**B**). The acquisition of luminescence images was performed as described in experimental part, using the Nightowl II imaging system (Berthold Technologies, Bad Wildbad, Germany). Data were expressed as photon/second reflecting the transfection efficiency of the different lipoplexes as mean ± SD (*n* = 3).

The luciferase reporter gene expression was measured by BLI 24h post injection for both lipoplexes. BLI was a simple and sensitive imaging technique that permitted the monitoring of luciferase expression in real time in living animals. Contrary to BFI, BLI did not require any excitation light. This technique allowed the determination of whether the targeted cells were transfected or not [4]. The bioluminescent images (Figure 6A) clearly showed a luciferase expression into the lungs, for pegylated and unpegylated fluorescent lipoplexes. This observation confirmed that the addition of fluorescent molecules into the lipophosphoramidate-based formulations did not affect their transfection properties. The transgene was predominantly expressed in the lungs, whereas a part of the injected complexes was also trapped in the liver and the spleen [47,48,49]. This was in agreement with the BFI observations presented above. In fact, the largest particles remained definitively blocked in the pulmonary microcirculation [50], and through a micro embolism phenomenon, induced the passage from vessels to epithelial cells [51]. However, a slight luciferase activity was also detected in the liver, probably due to the complexes trapped in this organ (Figure 5B). It was also probable that lipoplexes aggregates contained some very small particles able to escape from the lung microcirculation and passively reach other organs such as liver or spleen. The transfection efficiency of fluorescent lipophosphoramidate KLN47-based formulations was similar to that previously obtained by Le Gall *et al.* [13]. This means that addition of fluorescent dyes to lipophosphoramidate KLN47 did not affect its transfection properties. Quantification of luciferase expression at 24 h after injection showed a mean expression of 2.5 × 10^5^ ± 1.01 × 10^5^ photon/second for lipoplexes containing DSPE-PEG_2000_ and 4.22 × 10^5^ ± 7.13 × 10^4^ photon/second for unpegylated formulations (Figure 6B). Statistically, no significant difference (*p* = 0.09) was reported between both conditions (pegylated and unpegylated complexes). Indeed, it was previously demonstrated that the leakage rate of drugs from pegylated complexes was more rapid than that from unpegylated particles, which resulted in similar effects for both preparations [50]. The luciferase activity in lung tissue homogenates was very similar between pegylated and unpegylated formulations (data not shown). Thus, imaging with BLI and BFI were complementary. Indeed, BFI results informed about complex distribution, while the BLI data informed about the success of the transfection and the final transgene expression. However, we observed that while complexes could be located in an organ (BFI data), their physicochemical parameters, such as pegylation, and the nature of the tissue they were located in, could limit transfection capacities (BLI data). These observations were in agreement with previous reports established with other techniques [33,34].

#### 2.3.3. Biofluorescence Imaging in Isolated Organs

**Figure 7:**
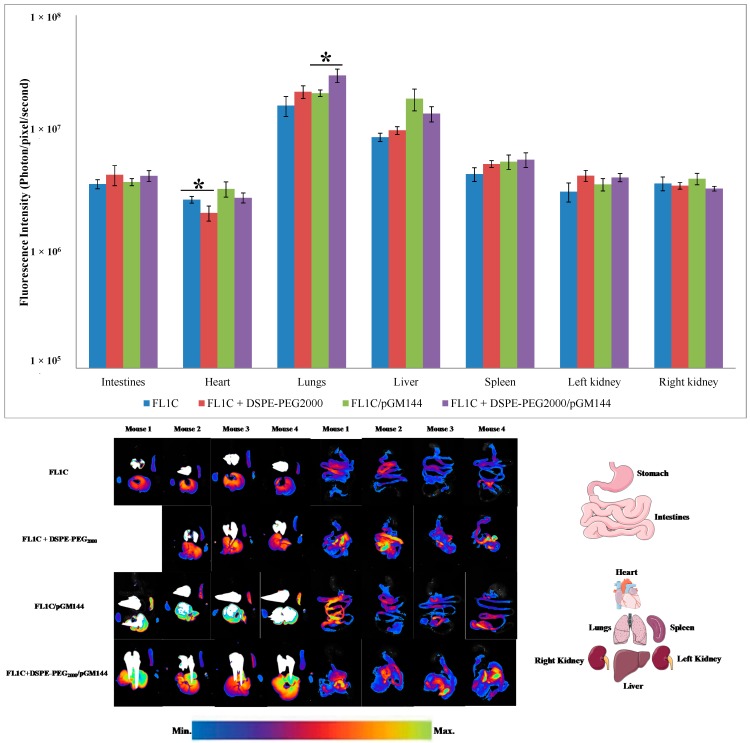
Biofluorescence of FL1C-based liposomes and lipoplexes from isolated organs after the systemic administration. Twenty-four hours post injection, mice were sacrificed by cervical dislocation and organs were collected for analysis. Fluorescence intensity was measured in different organs (lungs, liver, spleen, heart, intestines and kidneys). Results were expressed in photon/pixel/second obtained with each formulation reflecting the fluorescence intensity on different organs as mean ± SD (*n* = 4). * *p* < 0.05.

Twenty-four hours after the administration of the complexes, the animals were sacrificed by cervical dislocation and all their organs were collected for analysis. Fluorescent images in isolated organs were acquired as described in experimental section. Results showed clearly (Figure 7) that all organs were fluorescent at 24 h, principally the lungs and the liver (Figure 7). Mice receiving pegylated liposomes and lipoplexes showed a fluorescence accumulation principally in the lungs, the liver and to a lesser extent in the spleen. Indeed, complexes stabilized with DSPE-PEG_2000_ accumulated principally in the lungs (Figure 7) leading to risk of pulmonary embolism. In fact, particles presenting a larger size remained definitively blocked at alveolar capillaries. While the unpegylated complexes were essentially localized in the liver leading to their rapid elimination, the pegylation allowed escape from the first hepatic metabolism, prolonging the time that the particles resided in the blood circulation. These findings were confirmed by BFI images of isolated organs (Figure 7). ANOVA tests were performed to test the hypothesis of an existing difference between pegylated and unpegylated complexes. Results indicate that pegylated lipoplexes accumulated significantly in the lungs (*p* < 0.05). While the unpegylated lipoplexes were principally localized in the liver. In fact, the pulmonary system of humans and animals is composed of thin capillaries with a diameter ranging from 2 to 13 μm. Thus, only the particles having a diameter less than 3 μm pass through the pulmonary microcirculation to be distributed to other tissues. Those with a larger diameter remain permanently blocked at pulmonary level, thereby generating a risk of pulmonary embolism. This lung accumulation leads to a predominantly pulmonary transgene expression. Indeed, upon systemic administration, a large portion of the injected particles was trapped in the liver due to the liver first-pass metabolism leading to reduced bioavailability in the blood circulation. When the complexes were pegylated, they escaped to the hepatic first-pass metabolism. Consequently, their residence time and bioavailability in the bloodstream were prolonged. Concerning the unpegylated liposomes they were principally accumulated in the heart in comparison with the liposomes containing PEG (Figure 7). Gjetting *et al.* have reported that addition of 10% PEG to lipoplexes caused a reduced retention in heart tissues of nude mice in comparison with unpegylated lipoplexes [39]. These observations were in accordance with our previous BFI imaging (Figure 5A,B), and confirmed the benefit of pegylation to enhance the *in vivo* bioavailability of complexes [30,42,44].

#### 2.3.4. *In Vivo* Hepatotoxicity

**Figure 8:**
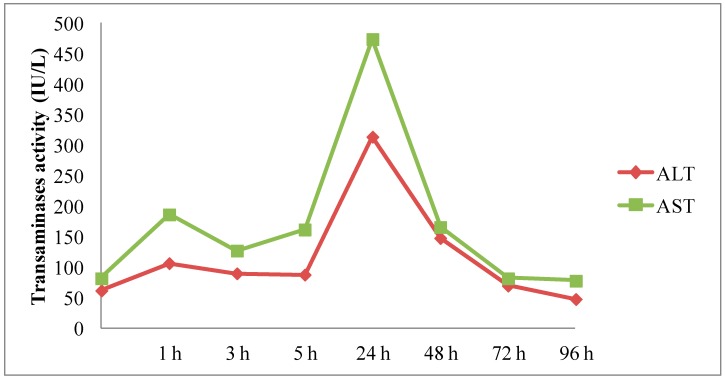
Kinetic of transaminase activities AST (green lines) and ALT (red lines) at different times following FL1C + DSPE-PEG_2000_/pGM144-based lipoplex administration. Results were based on one mouse per time, and expressed as IU/L.

To assess the physiological impact of complexes, blood samples were collected at different times after administration (from 1 to 96 h). Then, the liver injury was evaluated through the measurements of hepatic enzymes (AST: aspartate transaminase and ALT: alanine transaminase) from mice serum. Results were expressed as International Units per Liter (IU/L). As shown in Figure 8, systemic administration of FL1C + DSPE-PEG_2000_-based lipoplexes resulted in a rapid but transient increase in the transaminase activities a few hours after injection, reaching a peak at 24 h. Transaminases recovered their reference values 72 h after injection. Thus, systemic administration of pegylated fluorescent lipoplexes resulted in a transient liver toxicity. This transient hepatotoxicity was largely reported in the literature [21,26,27,52,53,54,55,56]. The AST/ALT transitory increase obtained with FL1C + DSPE-PEG_2000_/pGM144 was similar to AST/ALT profile obtained with unpegylated formulations [26]. In this previous study, our histological data reported that unpegylated lipoplexes confirm the induced hepatotoxicity profile [26]. The same hepatotoxicity profile was also obtained with many other DNA nanocarriers after systemic administration [27,37]. We assumed that the fluorescent compounds did not induce some additional liver toxicity, because the level of AST/ALT was very similar between both studies.

## 3. Experimental Section

### 3.1. Formulation of Fluorescent Cationic Lipids

In this study, we designed a new fluorescent-labeled lipid derivative containing a fluorescent dye compatible with *in vivo* imaging. Cyanine dye, *i.e.*, Cy3 carboxylic acid, was chosen as a fluorescent probe that possessed a carboxylic acid group which can be functionalized under mild and efficient conditions. To ensure anchoring of the probe into the liposome and lipoplex formulations, we synthesized a lipophosphoramidate with a primary amine group in the polar head region. Synthesis of the cyanine probe was performed according to procedures previously described by Simmons *et al.* [57]. Synthesis of the lipid anchor was achieved in one step from di-*O*,*O*-oleylphosphite and ethylenediamine under Atherton Todd conditions as described previously in our laboratory [19]. Thereafter, coupling of the lipid part with the cyanine dye was performed as peptide coupling reaction in the presence of EDC (*N*-(3-Dimethylaminopropyl)-*N*′-ethylcarbodiimide hydrochloride) as coupling reagent in a DMF (*N*,*N*-dimethylformamide)/THF (Tetrahydrofuran) (3/2) mixture due to solubility concerns. The fluorescent-labeled lipophosphoramidate 3 was isolated after flash column chromatography and was fully characterized by NMR and mass spectrometry analysis.

#### 3.1.1. General Methods

Compounds **1** and **2** were prepared from procedures previously described. Solvents were dried with a solvent purification system MBraun-SPS (THF, CH_2_Cl_2_) or freshly distilled on appropriate driers (DIPEA (*N*,*N*-Diisopropylethylamine) was distilled over NaOH). All compounds were fully characterized by ^1^H (500.13 or 400.133 or 300.135 MHz), ^13^C (125.773 or 75.480 MHz) and ^31^P (161.970 or 121.498 MHz) NMR spectroscopy (Bruker AC 300, Avance DRX 400 and Avance DRX 500 spectrometers). Coupling constants J are given in Hertz. The following abbreviations were used: s for singlet, d doublet, t triplet, q quadruplet, qt quintuplet, m for multiplet, dd for doublet of doublets and dt for doublet of triplets. When needed, ^13^C heteronuclear HMQC (Heteronuclear Multiple-Quantum Correlation) and HMBC (Heteronuclear Multiple-Bond Correlation) were used to unambiguously establish molecular structures. Mass spectrometry analysis was performed by on a Bruker Autoflex MALDI TOF-TOF III LRF200 CID (Academic RMN-RPE-SM Facilities, UBO, Brest, France). Fluorescence measurements were conducted on a Cary Eclipse Varian spectrophotometer. Other commercial compounds were used as received.

Synthesis of compound **1**: Compound **1** was synthesized as previously described by Mével *et al.* [19].

Synthesis of compound **2**: Compound **2** was synthesized as previously described by Simmons *et al.* [57].

**Scheme 1:**
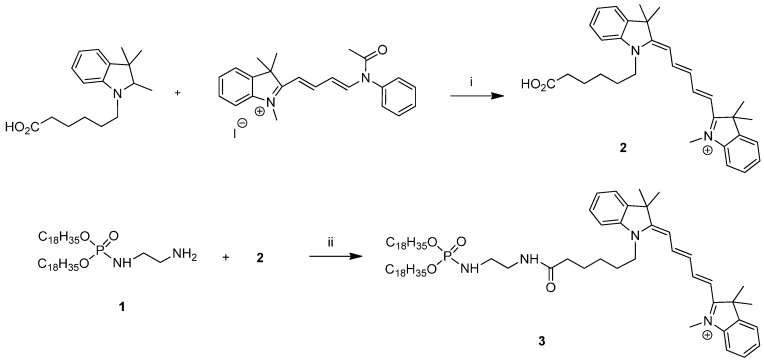
Synthesis of cyanine labeled lipophosphoramidate **3**. Reagents and conditions: i. Pyridine, 40 °C, 1.5 h; and ii. EDC, DMF/THF, RT, 18 h; Compound 1: *O*,*O*-dioleyl-*N*-ethylaminophosphoramidate, 2: 2-(5-(1-(5-Carboxypentyl)-1,3-dihydro-3,3-dimethyl-2H-indol-2-ylidene]-1,3-pentadienyl]-1,3,3-trimethyl-3H-indolium.

Synthesis of cyanine labeled lipophosphoramidate (Scheme 1). Compound **1** (100 mg, 0.16 mmol) and phosphoramide **2** (105 mg, 0.16 mmol) were combined in a dry DMF/THF mixture (3/2, 5 mL) under nitrogen. EDC (35 mg, 0.18 mmol) was added and the reaction was stirred at room temperature overnight. The reaction was diluted with DCM (Dichloromethane) (30 mL), washed with water, dried over MgSO_4_, filtered and concentrated. Column chromatography (DCM/MeOH 5%–10%) afforded the product as a dark blue solid (50 mg, 25% yield). **^1^H NMR** (400 MHz, CDCl_3_) : δ 0.89 (t, 3H), 1.21–1.35 (m, 44H), 1.54 (m, 4H), 1.58 (m, 4H), 1.68 (s, 12H), 1.78 (m, 4H), 1.97 (m, 8H), 3.14 (m, 2H), 3.38 (m, 2H), 3.69 (s, 3H), 3.93 (m, 4H), 3.97 (m, 2H), 4.57 (m, 1H), 5.29 (m, 4H), 6.32 (d, 1H, *J* = 14 Hz), 6.65 (d, 1H, *J* = 14 Hz), 7.01 (t, 1H, *J* = 14.0 Hz), 7.06 (d, 1H, *J* = 8.0 Hz), 7.17 (d, 1H, *J* = 8.0 Hz), 7.21–7.25 (m, 4H), 7.31–7.39 (m, 4H), 7.82 (q, 2H), 8.61 (m, 1H). **^31^P NMR** (400 MHz, CDCl_3_) : 10.5. **^13^C NMR** (MHz, CDCl_3_) 14.1, 22.7, 25.2–28.1, 28.2, 29.2–32.6, 36.2, 41.2, 41.6, 44.6, 49.0, 49.3, 66.5, 104.1, 104.6, 110.2, 110.9, 122.1, 125.0, 125.3, 127.0, 128.7, 129.7, 130.0–130.4, 140.7, 141.1, 141.9, 142.8, 152.6, 153.2, 172.6, 174.0. **HRMS** (Maldi-TOF) *m*/*z*: Calcd. for C_70_H_114_N_4_O_4_P: 1106.652 [M–H]^+^; Found: 1105.744.

#### 3.1.2. Absorption

Five diluted solutions of the compounds were prepared by successive dilutions of a stock solution. Absorption spectra were measured in 1-cm quartz cuvettes on a Cary 50 spectrophotometer and corrected by the solvent background. The maximum absorption wavelength, λ_max_, was determined and the absorbance at this wavelength was plotted *versus* the molar concentration, *c*. Absorbance values were in the range 0.05 to 0.5. A calibration line was obtained for each compound, according to the Beer-Lambert law:

*A =* ε_max_*l c*

The molar absorption coefficient at λ_max_, ε_max_, was calculated from the slope of the calibration line *A* = f (*c*) graph, taking *l* = 1 cm.

#### 3.1.3. Fluorescence

Fluorescence measurements were done on a Cary Eclipse Varian spectrofluorimeter (Agilent Technologies, Les Ulis, France) in 1-cm quartz cuvettes. An excitation-emission fluorescence matrix was first recorded in order to determine the main excitation-emission zone and also to check that only one fluorescent compound can be detected (the increment for excitation wavelength was 10 nm). Accurate excitation and emission spectra were then recorded (excitation and emission slits of 2.5 nm, slow scan) to determine excitation and emission maxima.

#### 3.1.4. Liposomes

Liposomes and fluorescent liposomes were prepared following the lipid film hydration method. Briefly, cationic lipid (KLN47) alone or with fluorescent cyanine containing lipid (5 mol %) was dissolved in chloroform. Solvent was evaporated to dryness under vacuum at 40 °C and the lipid thin film was further dried for 4 h. The lipid film was hydrated with sterile water (final concentration = mM) and stored at 4 °C overnight. Several concentrations were obtained (1.5, 11.5 and 23 mM) for *in vitro* and *in vivo* assays (Table 1). The lipid suspension was vortexed and sonicated for 30 min and liposomes size and zeta potential were measured from an aliquot of 50 μL after convenient dilution on a Malvern NanoZS apparatus (Malvern Instruments Ltd., Orsay, France).

### 3.2. In Vitro Experiments

#### 3.2.1. Plasmid DNA

The plasmid pGM144 (3.7 Kb) kindly provided from UK Cystic Fibrosis Gene Therapy Consortium (London, UK), encodes for the firefly luciferase protein under the control of the human 1 α elongation factor (hCEF1 α) promoter associated with the human CMV enhancer, and a CpG-free form of the replication origin R6K. This plasmid is entirely CpG-free in order to remove or reduce the inflammatory response associated to the C=G dinucleotides presence [28,29]. After amplification in *Escherichia coli* GT115 strain, the plasmids were extracted and purified using the endotoxin-free plasmid DNA (pDNA) purification kits (Macherey Nagel, Hoerdt, France) following the manufacturer’s instructions. The DNA concentration was determined by spectrophotometry at 260 nm (Hitachi-Sciences & Technologies, Berkshire, UK). Only plasmids with an A_260_/A_280_ ratio between 1.8 and 2 were used. The identity of the plasmid was confirmed by different enzymatic digestions followed by migration on 0.8% agarose gel.

#### 3.2.2. DNA Condensation Ability

Before the evaluation of the transfection efficiency, the ability of the synthetic vectors to complex pDNA was checked. This allowed the determination of the CR for which the NA was totally complexed. CR was defined as the number of positive charges carried by the cationic lipid relative to the number of negative charges carried by the DNA phosphates. With this goal, 1 µg of plasmid was mixed with corresponding cationic lipid to obtain CRs ranging from 1 to 8. All the formulations whatever their concentration, were evaluated. The complexes were prepared either in OptiMEM (Gibco, Villebon sur Yvette, France) for *in vitro* assays or in 0.9% NaCl for *in vivo* applications. The mixture was incubated for 1 hour at room temperature (RT). Then, 10 µL of each complex was deposited on 0.8% agarose gel. DNA retardation was assessed by electrophoresis (100 V, 90 mA during 30 min). The gel was stained with ethidium bromide (10 mg/mL) and visualized on UV transilluminator (Fischer Bioblock, Illkirch-Graffenstaden, France).

#### 3.2.3. *In Vitro* Transfection Assays

Preliminary tests were performed *in vitro* in order to evaluate the impact of the fluorescent probes on the KLN47-lipophosphoramidate formulations in term of transfection and toxicological impacts.

##### Cell Lines

*In vitro* assays were conducted on different epithelial cell lines: 16HBE (Human Bronchial Epithelial Cells), A549 (Adenocarcinoma Human Alveolar Basal Epithelial Cells CCL No. 2) and HeLa (Human Cervical Carcinoma CCL No. 185). A549 and HeLa cells were obtained from American-Type culture collection (Rockville, MD, USA). Considering the non-cancerous human bronchial epithelial cell line (16HBE), it was kindly provided by Dieter Gruenert (University of San Francisco, CA, USA) They were grown in EMEM (16HBE), or DMEM (A549 andHeLa), supplemented with 10% bovine fetal serum, 1% antibiotic and 1% l-Glutamine, in a humidified incubator with 5% CO_2_ at 37 °C.

##### Transfection Procedure

Cells were seeded 24 h before transfection onto a 96-well plate, at a density of 25,000 cells per well, in 200 µL of their specific medium and incubated overnight in a humidified incubator with 5% CO_2_ at 37 °C. Each condition was tested in triplicate. Transfection was performed as following described. Various lipoplexes characterized by several CRs (1 to 8) were prepared with a constant amount of DNA plasmid (0.25 µg) in OptiMEM medium (Gibco). Naked pDNA, untransfected cells and the commercial Lipofectamine 2000 (LFM, Invitrogen, Saint Aubin, France) were used as controls. After 1 h of incubation at RT, 30 µL of each lipoplex was added to the corresponding well. The plates were then incubated for 48 h until revelation.

##### Reporter Gene Expression

Forty-eight hours after transfection, cells were assayed for reporter gene expression using a chemiluminescent assay. After removing culture medium, 50 µL of 0.5X Passive Lysis Buffer (PLB, Promega, Charbonnières-les-Bains, France) were deposited in each well, and the plates were frozen at −20 °C. Then, plates were thawed at RT. After thawing, 25 µL was taken from each well, and transferred to a white 96-well plate. The luciferase activity in the supernatant was quantified using a luminometer (MLX^®^ Microtiter Plate Luminometer-Dynex, Guyancourt, Paris, France). The 25 µL remaining in the starting plate was used to determine the protein concentration, using the photometric method with bicinchoninic acid (BCA protein assay reagent kit, Interchim, Montluçon, France). In parallel, a standard range of Bovine Serum Albumin (BSA, Interchim, Montluçon, France) was performed to associate each absorbance value measured with a protein concentration. Two hundred µL of reconstituted reagent was added to each well, and then the plates were incubated at 37 °C for 30 min. The protein concentration in each well was then measured at 540 nm (spectrophotometer ELx808™, BioTek Instruments, Fisher Scientific, Illkirch, France). The data were expressed as RLU per mg of total protein (RLU/mg of total proteins).

##### Cytotoxicity Assessment

Evaluation of cytotoxicity was performed using the Toxilight Bioassay kit (Lonza, Verviers, Belgique). This kit was based on the activity of AK that was normally present exclusively in the cytoplasm. Cell membrane alterations resulted in the release of AK enzyme in the culture medium, revealing the cytotoxicity induced by the formulations. Two hours after the deposit of complexes onto the cells, 20 µL of supernatant was collected per well, to estimate the early cytotoxicity. This assay was carried out, as specified by the manufacturer, and the results were expressed as RLU. The RLU were conversely proportional to the intensity of damages induced to the cells. Untransfected cells were used to evaluate the AK standard level and were considered as the reference in term of toxicity.

### 3.3. In Vivo Experiments

#### 3.3.1. *In Vivo* Systemic Administration

*In vivo* biodistribution assays were conducted with female NMRI nude mice of 6 weeks (JANVIER Rodent research models & associated services LABS, Saint Berthevin, France). This project was approved by the ethic committee of Finistère No: 74 and registered under the reference 00567.01., from 26 May 2014 to 21 July 2014. High concentrated FL1C formulation was used for *in vivo* applications. Such formulation permitted to inject a low volume of compounds. Mice were housed and maintained at the university animal facility; they were processed in accordance with the Laboratory Animal Care Guidelines and with the agreement of the regional veterinary service. It was previously demonstrated that CR = 4 was generally efficient both in *in vitro* and *in vivo* assays [13,26]. Then, appropriate quantities of pGM144 and FL1C were mixed and 200 µL was injected to each mouse at CR of 4 containing 50 µg of pDNA, in presence or absence of DSPE-PEG_2000_ (Avanti^®^ Polar Lipids, Inc. COGER, Paris, France). We used DSPE-PEG_2000_ in order to protect and to enhance the circulation time of complexes [42]. FL1C based lipoplexes or liposomes ± DSPE-PEG_2000_ were prepared in 0.9% NaCl for *in vivo* assays. After 1 hour of incubation at RT, 200 µL of complexes was injected via the tail vein to each mouse.

#### 3.3.2. *In Vivo* Biofluorescence Imaging

Non-invasive BFI was performed successively at 1, 3, 5 and 24 h post injection, using the BFI system of the Nightowl II (Berthold Technologies, Bad Wildbad, Germany). This system was equipped with a cooled, slow-scan CCD camera and driven with the Winlight32 software (Berthold Technologies, Bad Wildbad, Germany). Considering the fluorescent characteristics of cyanine, we selected the 630 nm excitation and 655 nm emission filters. BFI images were taken onto different positions to obtain general view of fluorescence distribution.

The animals were previously anesthetized with a 4% air-isoflurane blend. They were maintained anesthetized with a 2% air-isoflurane mixture all along the acquisition, in the acquisition chamber (Berthold Technologies, Bad Wildbad, Germany). The fluorescent acquisition time was 1 second and the fluorescent signal was then overlaid on a picture of corresponding mouse. Results were expressed as photon/pixel/second.

#### 3.3.3. *In Vivo* Bioluminescence Imaging

In order to evaluate the transfection efficiency of the fluorescent cationic lipid, non-invasive BLI was performed 24 h post-injection of lipoplexes, using the *luc* reporter gene system. First, mice received by intraperitonial injection 200 µL of luciferin (20 mg/mL in 20 mM Hepes buffer; Luciferins, FluoProbesR, Interchim, Montluçon, France). Then, they were anesthetized as previously described. Luminescence images were performed using the Nightowl II imaging system (Berthold Technologies, Bad Wildbad, Germany) associated with the WinLight 32 software (Berthold Technologies, Bad Wildbad, Germany), with a binning of 8 × 8 and an exposure time of 4 min. Luminescence images were then overlaid onto images of each corresponding mouse. The luminescence intensities were quantified as photon/second.

#### 3.3.4. Biofluorescence Imaging in Isolated Organs

Twenty-four hours after complex administration, the animals were sacrificed by cervical dislocation and all their organs were collected for fluorescence analysis. Fluorescent images in isolated organs were acquired as previously described for the whole animal, and results were presented in photon/pixel/second.

#### 3.3.5. *In vivo* Hepatotoxicity Assessment

To assess the physiological impact of administered complexes, blood samples were collected from each mouse at different times post administration (from 1 to 96 h). Around 100 to 200 µL were collected from the saphenous vein of treated mice on a heparin tube. Then, tubes were centrifuged at 10,000 g for 2 min at 4 °C and blood plasma were collected to measure ALT and AST levels, using a commercial kit (Elitech, Puteaux, France) and according to the provider recommendations. This method was based on the absorbance kinetic at 340 nm corresponding to the oxidation of NADH into NAD when transaminases were in contact with their substrates. Results were expressed as International Units per Liter (IU/L).

### 3.4. Statistical Test

The Student *t*-Test and ANOVA test were used to statistically compare the transfection efficiency of pegylated and unpegylated complexes. A *p* value of less than 0.05 was considered significant.

## 4. Conclusion

The study presented here explored the biodistribution profiles of new lipidic fluorescent complexes following IV administration in nude mice. Data showed that labelling lipophosphoramidates with fluorescent probes did not affect their properties in terms of gene delivery ability and tolerance. Results highlighted the potential of these molecules as tracers of lipophosphoramidate-based liposomes or lipoplexes to investigate *in situ* biodistribution of the particles as well as cell trafficking. This work demonstrated also the duality of the pegylation, providing a high bioavailability, but limiting transfection capacity. Such multimodular synthetic systems could be used to guide the formulation of complexes and some new fluorescent compounds will be explored in the near future [35] using FRET (Förster Resonance Energy Transfer) activity.

## Supplementary Materials

Supplementary materials can be found at http://www.mdpi.com/1422-0067/16/11/25941/s1.

## Abbreviations

AK: Adenylate kinase; ALT: Alanine Transaminase; AST: Aspartate Transaminase; BFI: BioFluorescence Imaging; BLI: Bioluminescence Imaging; CR: Charge Ratio; DCM: Dichloromethane; DIPEA: *N*,*N*-Diisopropylethylamine; DMF: *N*,*N*-dimethylformamide; DSPE-PEG_2000_: 1,2-Distearoyl-sn-Glycero-3-phosphoethanolamine-*N*-[Methoxy(Polyethyleneglycol)-2000] (Ammonium salt); EDC: *N*-(3-Dimethylaminopropyl)-*N*′-ethylcarbodiimide hydrochloride; FL: Fluorescent Lipid; HMBC: Heteronuclear Multiple-Bond Correlation; HMQC: Heteronuclear Multiple-Quantum Correlation; IV: Intravenous injection; LFM: Lipofectamine; MeOH: Methanol; NA: Nucleic Acids, NBD: 7-Nitrobenz-2-oxa-1,3-diazol-4-yl; PEG: PolyEthylene Glycol; RLU: Relative Light Units; and RT: Room Temperature, and THF: Tetrahydrofuran.
