# Supplementary material for: Evaluation of New Fluorescent Lipophosphoramidates for Gene Transfer and Biodistribution Studies after Systemic Administration
Source: Int J Mol Sci. 2015 Nov 2;16(11):26055–76. doi: 10.3390/ijms161125941 (PMC4661800; doi:10.3390/ijms161125941)
Supplement: Supplementary file 1 [file ijms-16-25941-s001.pdf]

International Journal of
Molecular Sciences
MDPI

Supplementary Information

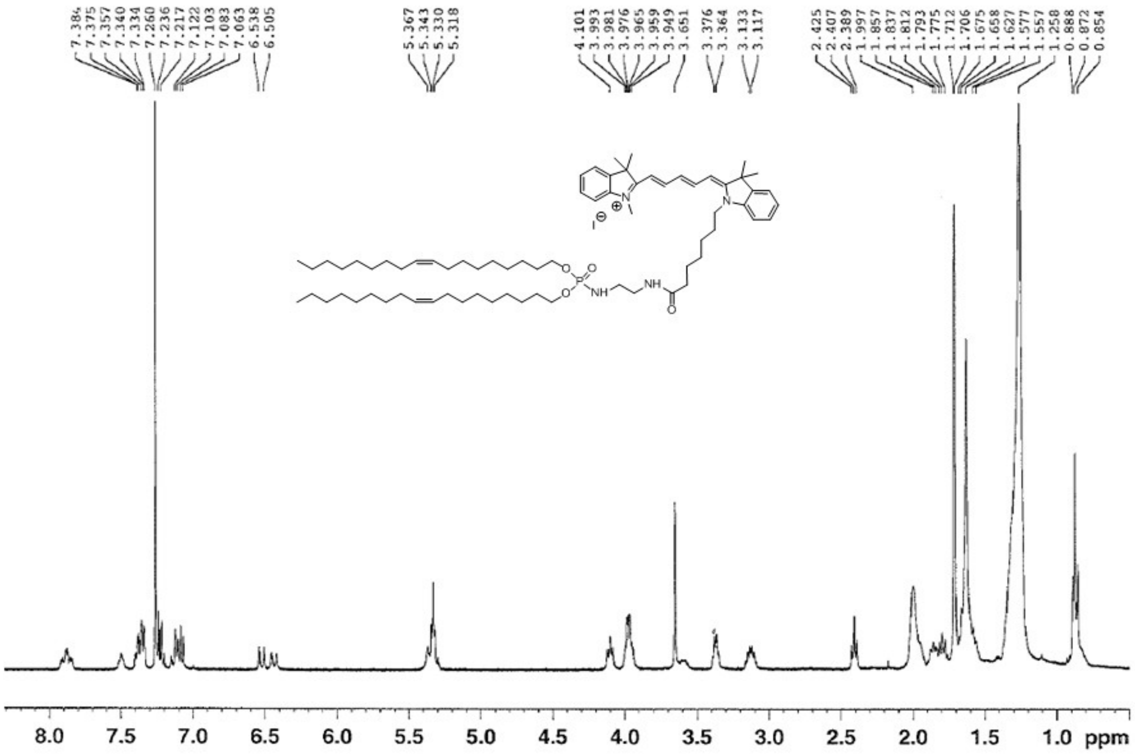

^{1}H-NMR spectrum of compound **3**

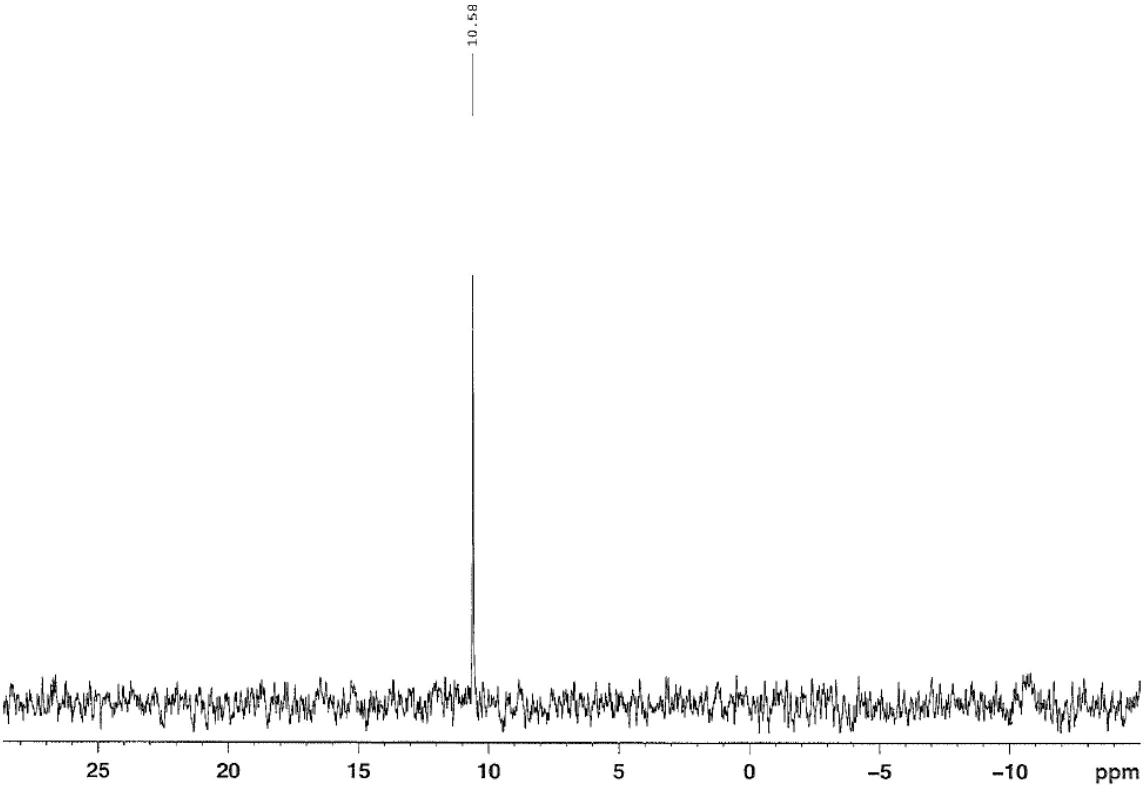

^{31}P NMR spectrum of compound **3**

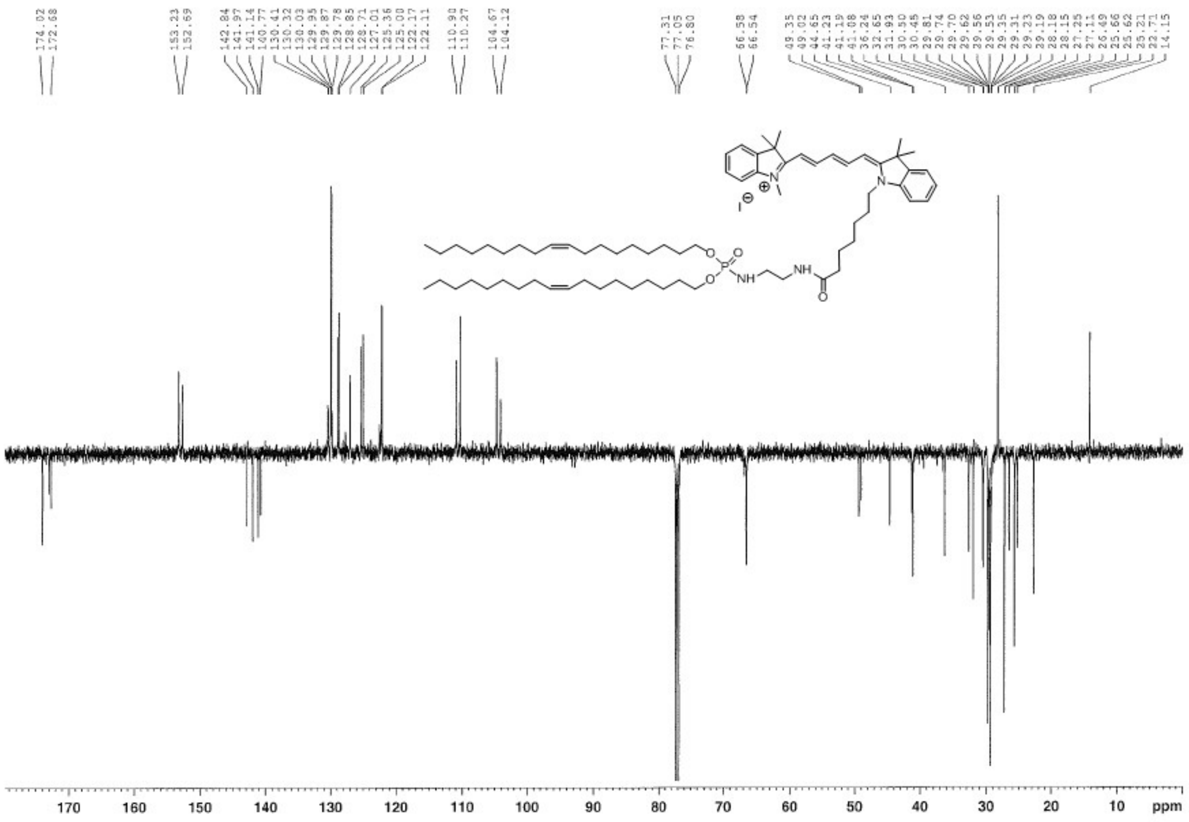

^{13}C NMR spectrum of compound **3**

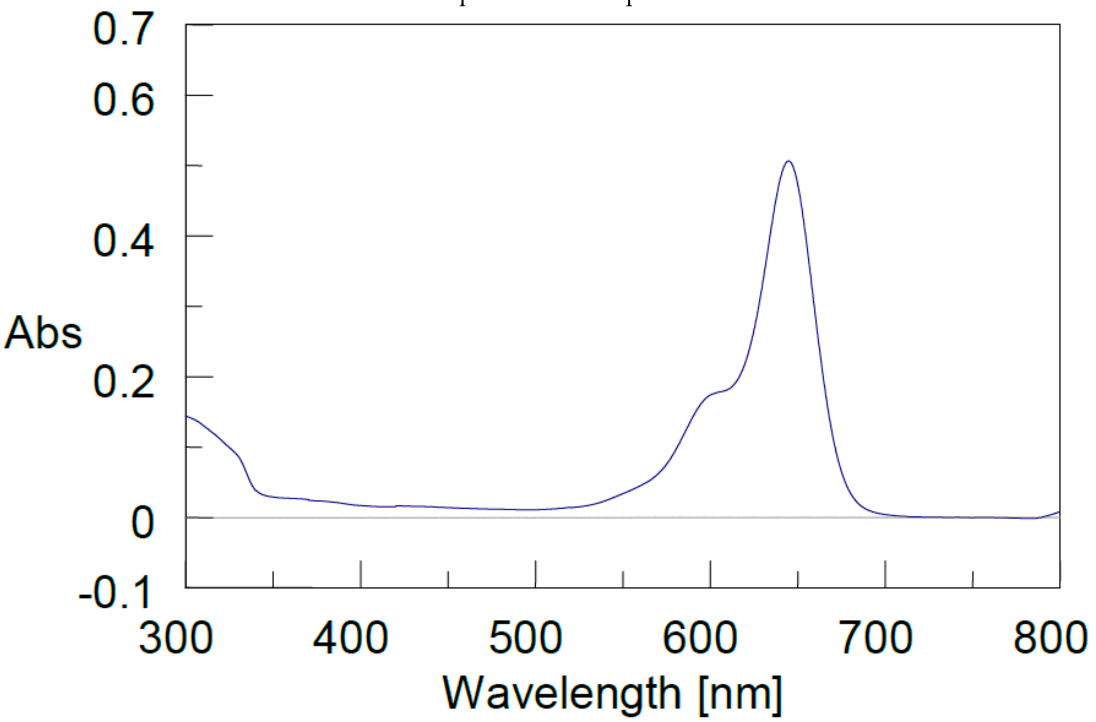

Absorbance and fluorescence spectrum of compound **3**

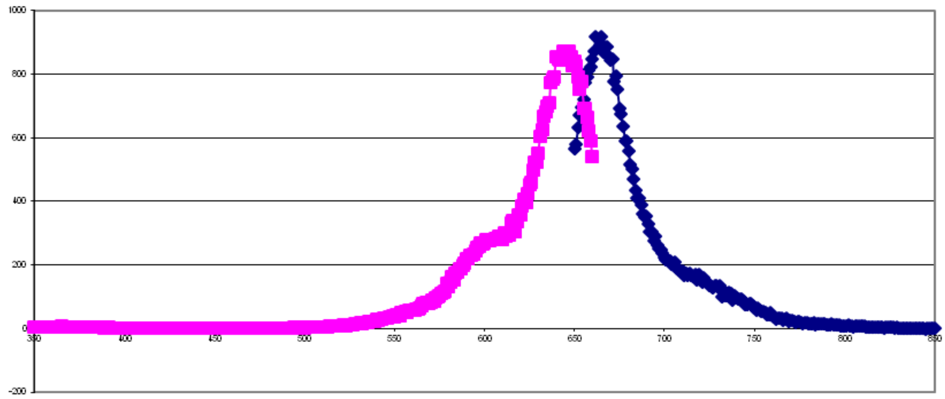

Fluorescence spectrum of compound **3**

Scheme S1. NMR, absorbance and fluorescence spectra compound **3**.
